# Is Ultra-Short-Term Heart Rate Variability Valid in Non-static Conditions?

**DOI:** 10.3389/fphys.2021.596060

**Published:** 2021-03-30

**Authors:** Jin Woong Kim, Hyeon Seok Seok, Hangsik Shin

**Affiliations:** Department of Biomedical Engineering, Chonnam National University, Yeosu-si, South Korea

**Keywords:** autonomic nervous system, heart rate variability, mobile healthcare, ultra-short-term heart rate variability, electrocadiogram

## Abstract

In mobile healthcare, heart rate variability (HRV) is increasingly being used in dynamic patient states. In this situation, shortening of the measurement time is required. This study aimed to validate ultra-short-term HRV in non-static conditions. We conducted electrocardiogram (ECG) measurements at rest, during exercise, and in the post-exercise recovery period in 30 subjects and analyzed ultra-short-term HRV in time and frequency domains by ECG in 10, 30, 60, 120, 180, and 240-s intervals, and compared the values to the 5-min HRV. For statistical analysis, null hypothesis testing, Cohen’s *d* statistics, Pearson’s correlation coefficient, and Bland-Altman analysis were used, with a statistical significance level of *P* < 0.05. The feasibility of ultra-short-term HRV and the minimum time required for analysis showed differences in each condition and for each analysis method. If the strict criteria satisfying all the statistical methods were followed, the ultra-short-term HRV could be derived from a from 30 to 240-s length of ECG. However, at least 120 s was required in the post-exercise recovery or exercise conditions, and even ultra-short-term HRV was not measurable in some variables. In contrast, according to the lenient criteria needed to satisfy only one of the statistical criteria, the minimum time required for ultra-short-term HRV analysis was 10–60 s in the resting condition, 10–180 s in the exercise condition, and 10–120 s in the post-exercise recovery condition. In conclusion, the results of this study showed that a longer measurement time was required for ultra-short-term HRV analysis in dynamic conditions. This suggests that the existing ultra-short-term HRV research results derived from the static condition cannot applied to the non-static conditions of daily life and that a criterion specific to the non-static conditions are necessary.

## Introduction

The measurement of heart rate variability (HRV) is used to assess autonomic nervous system (ANS) activity and is known to be meaningful in assessing cardiac vagal activity (Malik et al., 1996; Berntson et al., 1997; Laborde et al., 2017). HRV measurement analyzes variations in the inter-beat interval seen in the electrocardiogram (ECG). In general, long-term HRV is derived from a 24 h ECG, and short-term HRV is derived from a 5 min ECG (Camm et al., 1996). While long-term recording has the potential to analyze physiological statuses, such as congestive heart failure, mitral regurgitation, and mortality (Camm et al., 1996), short-term HRV is becoming more popular because long-term HRV is difficult to measure in everyday life. Recently, for monitoring the health situations of an individual in daily life, the use of HRV has greatly increased in connection with mobile and wearable technologies. However, when used for routine healthcare purposes, the subject may feel that even the 5 min steady-state measurement required for short-term HRV analysis is long, which may result in measurement discomfort. Moreover, real-time measurements are difficult while the human body is in motion, and are likely to yield inaccurate results. To overcome these practical limitations and increase the usefulness of HRV in mobile and wearable situations, ultra-short-term HRV with an analysis interval of less than 5 min has been studied (Hamilton et al., 2004; Flatt and Esco, 2013; Castaldo et al., 2017). Previous studies have calculated HRV at intervals between 10 s and 10 min, the minimum time required to identify statistically significant differences for each ultra-short-term HRV variable compared to short-term HRV, as summarized in Table 1. While the previous results may seem to suggest the validity of ultra-short-term HRV, they cannot be generalized because each variable had a very large variation, which depended upon the analysis method or condition.

**TABLE 1:** Minimum time requirements for ultra-short-term HRV analysis.

**HRV variable**	**Minimum time requirements for ultra-short-term HRV analysis**	**References**
**Time domain**		
Average of normal-to-normal interval (AVNN)	10 s	McNames and Aboy, 2006; Salahuddin et al., 2007; Nussinovitch et al., 2011; Baek et al., 2015
Standard deviation of normal-to-normal interval (SDNN)	30–240 s	McNames and Aboy, 2006; Nussinovitch et al., 2011; Baek et al., 2015; Munoz et al., 2015
Root-mean-square of successive difference (RMSSD)	10–30 s	McNames and Aboy, 2006; Salahuddin et al., 2007; Nussinovitch et al., 2011; Baek et al., 2015; Munoz et al., 2015
The percentage of adjacent NN intervals that differ from each other by more than 50 ms (pNN50)	30–60 s	Salahuddin et al., 2007; Baek et al., 2015
**Frequency domain**		
Total power (TP)	240 s	Baek et al., 2015
Very-low-frequency power (VLF)	270 s	Baek et al., 2015
Low-frequency power (LF)	40–250 s	Malik et al., 1996; Salahuddin et al., 2007; Baek et al., 2015
High-frequency power (HF)	20–180 s	Malik et al., 1996; Salahuddin et al., 2007; Baek et al., 2015
The ratio of low-frequency power to high-frequency power (LF/HF)	50–90 s	McNames and Aboy, 2006; Salahuddin et al., 2007; Baek et al., 2015
Normalized low-frequency power (nLF)	50–90 s	Salahuddin et al., 2007; Baek et al., 2015
Normalized high-frequency power (nHF)	50–90 s	Salahuddin et al., 2007; Baek et al., 2015

The biggest pitfall of the previous studies was that ultra-short-term HRV was only evaluated in non-dynamic conditions, such as resting, despite its suggested use in situation including mobile healthcare. Dynamic activities occurring in daily life can activate and inhibit the autonomic nervous system, which can greatly affect HRV. Previous studies demonstrated that HRV was a reliable tool for assessing autonomic control of the HR during dynamic conditions such as walking before and after maximal effort (Hunt and Saengsuwan, 2018). However due to the exercise performed, average of normal-to-normal intervals (AVNN), standard deviation of normal-to-normal intervals (SDNN), root-mean-square of successive difference (RMSSD), percent of successive difference of normal-to-normal interval exceeds 50 ms (pNN50), total power (TP), low frequency (LF), high frequency (HF), and very low frequency (VLF) changed significantly from the resting state (Javorka et al., 2002; Leicht et al., 2008). In addition, significant changes in the SDNN, RMSSD, LF, and HF were observed according to exercise intensity (Buchheit et al., 2007; Martinmäki et al., 2008). Changes in HRV in post-exercise recovery conditions were also reported (Barak et al., 2010; Stanley et al., 2013). The above results demonstrate that the dynamic state causes a change in HRV, which may have a big impact on the analysis interval needed for ultra-short-term HRV. Thus, a separate analysis and dedicated criteria for ultra-short-term HRV in dynamic conditions are essential.

In the mobile healthcare environment, HRV is measured not only in the resting state, but also in various dynamic states such as standing, moving, and stopping. In addition, due to the nature of dynamic state measurements, a measurement time as short as possible is required. This study aimed to examine the feasibility of ultra-short-term HRV measurements in steady-state conditions, as well as dynamic conditions, using various statistical techniques. To this end, ultra-short-term HRV analysis was performed using ECGs obtained during rest, while exercising, and during post-exercise recovery, and the minimum required time for ultra-short-term HRV analysis was investigated in each condition and compared to the short-term HRV results.

## Materials and Methods

### Exercise Protocol

**FIGURE 1:**
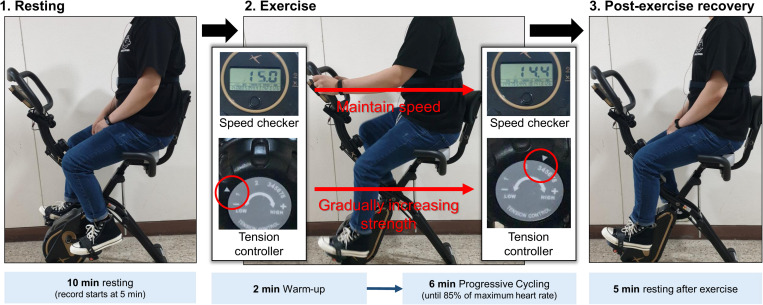
Experimental protocol.

The experiments in this study were performed in the order of resting, while performing progressive resistance exercises, and during post-exercise recovery to induce autonomic activation in each condition (Figure 1). To minimize motion artifacts due to upper body movement during exercise, a stationary bicycle, DP-652-G-1 (Iwhasmp Inc., Seoul, South Korea) was used. The stationary bicycle used in the study had a total of eight levels of exercise intensity. Steps 1 and 2 were set to warm-up, steps 3, 4, 5, and 6 were set to moderate-intensity training, and steps 7 and 8 were set to high-intensity training. In the resting stage, the subject took a 10-min rest in the sitting position, and the latter 5 min of the ECG was used for HRV analysis. The exercise was performed for a total of 8 min, including 2 min of warm-up, taking into account the recommended exercise time for cardiopulmonary exercise testing (CPET) (Datta et al., 2015). During the exercise, the speed was maintained at 14–16 m/s. In the warm-up phase, the tension controller of the stationary bicycle was set to step one. After that, the step of the tension controller was increased by one step every two min to gradually increase the exercise intensity. In general, a heart rate greater than 85% of the age-predicted maximal heart rate is defined as high-intensity exercise, so when the subject’s heart rate reached 85% of the maximum age-related heart rate, the subject immediately stopped exercising and rested (Fletcher et al., 2013). Measurements in the post-exercise recovery condition were performed with the subject sitting comfortably on the stationary bicycle for 5 min. To exclude factors affecting HRV, subjects who had been drinking alcohol, consuming caffeine, smoking, had a lack of sleep, or were taking medication within 24 h that could affect the autonomic nerves were excluded. Since autonomic nervous system activity can change according to temperature and humidity (Zhu et al., 2018), the humidity of the measurement space was maintained at 65% and the temperature was maintained at room temperature at (20–28°C). The proposed protocol was approved by the Institutional Review Board (IRB) of Chonnam National University (IRB No. 1040198-190821-HR-090-02, Gwangju, South Korea). Figure 1 shows the experimental protocol and environment. All subjects provided written informed consent.

### Data Recording and Signal Preprocessing

**FIGURE 2:**
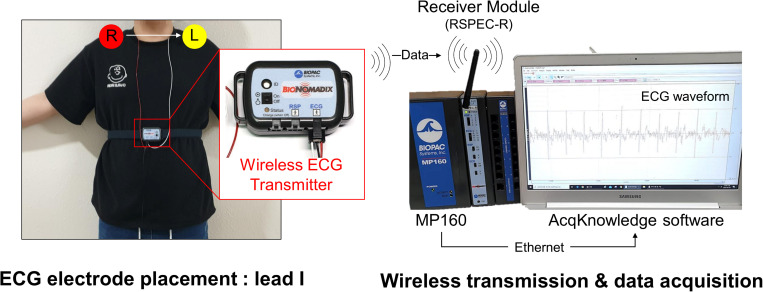
System configuration for obtaining electrocardiograms.

**FIGURE 3:**
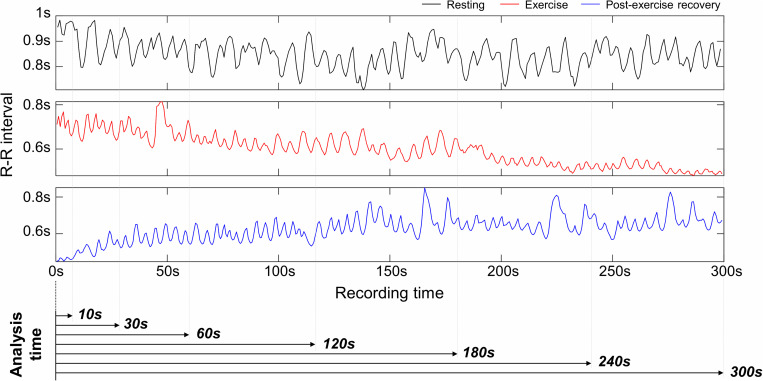
Examples of R–R interval in resting, exercising, and post-exercise recovery conditions, and analysis intervals for ultra-short-term heart rate variability analysis.

The experimental protocol lasted a total of 23 min and ECG recordings were made for 18 min, except for the initial 5 min of the resting stage. ECG was measured with lead I at 1 kHz sampling frequency. An MP150 (BIOPAC Systems, Inc., CA, United States) and wireless module RSPEC-R (BIOPAC Systems, Inc., CA, United States) were used for the ECG measurements (Figure 2). The measured ECGs were stored on a laptop using BIOPAC’s AcqKnowledge software. As exercise can change the R–R interval (RRI) rapidly during warm-up, only the progressive cycling data after warm-up were used. ECG was bandpass filtered with a 0.05–30 Hz passband, and the QRS-complex was detected by the Pan and Tompkins (1985) QRS detection algorithm (threshold = 0.2). Experienced researchers checked whether the QRS complex was appropriately detected and manually corrected falsely detected QRS complex. Prior to data analysis, pre-screening was conducted and cases where the QRS waveform could not be intuitively observed due to severe motion artifacts were excluded from the analysis. During measurement, instrument errors and severe subject movements may cause errors in QRS detection such as a mis-detected QRS or missing QRS, which may result in abnormal RRIs. For example, a falsely detected QRS complex decreases or increases the RRI, and a missed QRS complex prolongs the RRI. Thus, the abnormal range of RRIs were corrected by removing and interpolating when the current RRI increased by more than 32.5% or decreased by more than 24.5% from the previous RRI (Marked, 1995; Choi and Shin, 2018). Linear interpolation was used for interpolating the RRIs. To analyze HRV according to the analysis time, the analysis interval was set to 10, 30, 60, 120, 180, 240, and 300-s lengths (Figure 3). After calculating the RRI for each analysis interval, time domain and frequency domain HRV variables were derived. As a result of the HRV analysis, seven resting condition datasets, seven exercise condition datasets, and seven post-exercise recovery condition datasets were derived per subject, including six ultra-short-term HRVs per measurement condition.

### HRV Parameters

The HRV variables used in this study were AVNN, SDNN, standard deviation of successive difference (SDSD), RMSSD, and pNN50 in the time domain, and TP, VLF, LF, HF, LF/HF, normalized LF (nLF), and normalized HF (nHF) in the frequency domain (Acharya et al., 2006). Table 2 shows the definition of each HRV variable. For the HRV frequency domain analysis, the RRIs were transformed to an evenly sampled time series by resampling with 4 Hz after 1 kHz linear interpolation, and both the mean and linear trends were removed. The power spectral density was estimated by fast Fourier transform. The frequencies corresponding to VLF, LF, and HF were set to 0.0033–0.04, 0.04–0.15, and 0.15–0.4 Hz, respectively. HRV variables reflect various physiological activities related to the autonomic nervous system. The HF reflects parasympathetic or vagal activity frequently called the respiratory band. The LF is known to primarily reflect baroreceptor activity while at rest (Malliani, 1995).

**TABLE 2:** Definition of HRV variables.

**HRV variables**	**Unit**	**Description**
**Time domain**		
AVNN	ms	Average of all NN intervals
SDNN	ms	Standard deviation of all NN intervals
SDSD	ms	Standard deviation of differences between adjacent NN intervals
RMSSD	ms	The square root of the mean of the sum of the squares of differences between adjacent NN intervals
pNN50	%	NN50 count divided by the total number of all NN intervals
**Frequency domain**		
TP	ms^2^	The variance of NN intervals over the temporal segment
VLF	ms^2^	Power in the very-low-frequency range
LF	ms^2^	Power in the low-frequency range
HF	ms^2^	Power in the high-frequency range
LF/HF	n.u.	LF power in normalized units
nLF	n.u.	HF power in normalized units
nHF	n.u.	LF/HF ratio

*n.u., null unit.*

Since this study aimed to verify the significance of short-term and ultra-short-term HRVs, the SDNN index (the mean of the 5 min standard deviation of the average NN intervals), the standard deviation of the average NN intervals (SDANN), and ultra-low-frequency power (ULF) were excluded from the analysis.

### Statistical Analysis

Statistical significance was assessed for each variable of the ultra-short-term HRV and short-term HRV measured at rest, while exercising, and during post-exercise recovery. Prior to the analyses, all HRV variables were log-transformed to obtain approximately normal distributions. For statistical analysis, to test agreement with the null hypothesis, the ANOVA test was performed when the equivariance and normality conditions were satisfied, and the Kruskal-Wallis test was performed when the equivariance was satisfied without normality. Otherwise, Friedman’s test was performed. Bonferroni’s post hoc test was carried out for inter-group comparisons. Prior to the analysis of variance, Levene’s test was used to assess the homogeneity of variance, and the Kolmogorov-Smirnov test was used to test normality. *P*-values of less than 0.05 were considered to indicate statistical significance.

We calculated Cohen’s *d* statistics to quantify the bias of the HRV measurements of different analysis intervals relative to their within-group variations (Cohen, 1988). Cohen’s *d* is an appropriate effect size indicating the standardized measure of the size of the mean difference or the relationship among the study groups and is used to indicate the standardized difference between two means. Cohen’s *d* is determined by calculating the mean difference between the two groups and then dividing the result by the pooled standard deviation that is a weighted average of the standard deviations of two or more groups. The individual standard deviations are averaged, with more weight given to larger sample sizes (Equation 1).

(1) $${\mathrm{Cohen}}^{\prime }\,s\,d=\frac{\left(M_{2}-M_{1}\right)}{{\text{SD}}_{\text{pooled}}}$$

where, SD_pooled_ is $\sqrt{\left({\text{SD}}_{1}^{2}+{\text{SD}}_{2}^{2}\right)\,/\,2}$, and *M*_1_ and *M*_2_, and SD_1_ and SD_2_ are the mean and standard deviation of the two groups, respectively.

The *d* value is interpreted in a range from 0.01 to 2.0, where 0.01 is very small, 0.2 is small, 0.5 is medium, 0.8 is large, 1.2 is very large, and 2 is huge (Cohen, 1988; Rosenthal, 1990). A *d* of 1 indicates that the two groups differ by 1 standard deviation, while a *d* of 2 indicates that they differ by 2 standard deviations.

In this study, a *d* value of less than 0.5 was set as the criterion indicating two groups had similar values, and the minimum required interval was evaluated using shortest ultra-short-term HRV interval measured as the minimum time interval for which this criterion was maintained. Pearson’s correlation coefficients (*R*) were calculated between the short-term HRV and the ultra-short-term HRV values. In this study, *R* > 0.8, which is generally used to represent a strong correlation, was set as the ultra-short-term HRV measurability criterion. However, a strong correlation does not guarantee a close agreement between two groups. Therefore, the Bland–Altman (BA) analysis was carried out to analyze the agreement between short-term HRV and ultra-short-term HRV with 95% limits of agreement (LoA) (Altman and Bland, 1983; Bland and Altman, 2003). We performed a BA analysis with the *x*-axis as the ground truth (short-term HRV) (Krouwer, 2008). The bias was calculated as the mean difference between the short-term HRV and ultra-short-term HRV measurements. In this study, we examined whether the LoA of the ultra-short-term HRV variable included the zero-difference line of the BA plot, indicating the short-term HRV equaled the ultra-short-term HRV. This is not the method used in the general BA analysis but was used to check the measurability based on whether the LoA of the estimated value of the ultra-short-term HRV included the ground truth. We used 50% LoA as a decision threshold, which means that the interquartile range of the derived ultra-short-term HRV variable included the ground truth. Matlab 2016a (Mathworks, Inc., MA, United States) was used for signal processing in the HRV analysis and statistical analyses.

## Results

### Experimental Data

Experiments and data acquisition were performed on 30 healthy adults without cardiovascular disease, eight of whom were excluded due to severe motion artifacts during the experiment, preventing the QRS from being distinguished intuitively. Finally, the data from 22 participants were used for the analysis. Table 3 shows the demographics of the subjects who participated in the experiment.

**TABLE 3:** Participant demographics.

**Sex (N)**	**Age (years)**	**Height (cm)**	**Weight (kg)**	**Body mass index (kg/m^2^)**
Male (15)	25.5 ± 4.5	175.1 ± 3.9	71.2 ± 11.5	23.1 ± 3.3
Female (7)	21.5 ± 1.9	159.4 ± 1.9	54.8 ± 4.2	21.2 ± 2.0
Total (22)	24.2 ± 4.3	170.1 ± 8.0	66.0 ± 12.4	22.5 ± 3.1

### Null Hypothesis Testing

**FIGURE 4:**
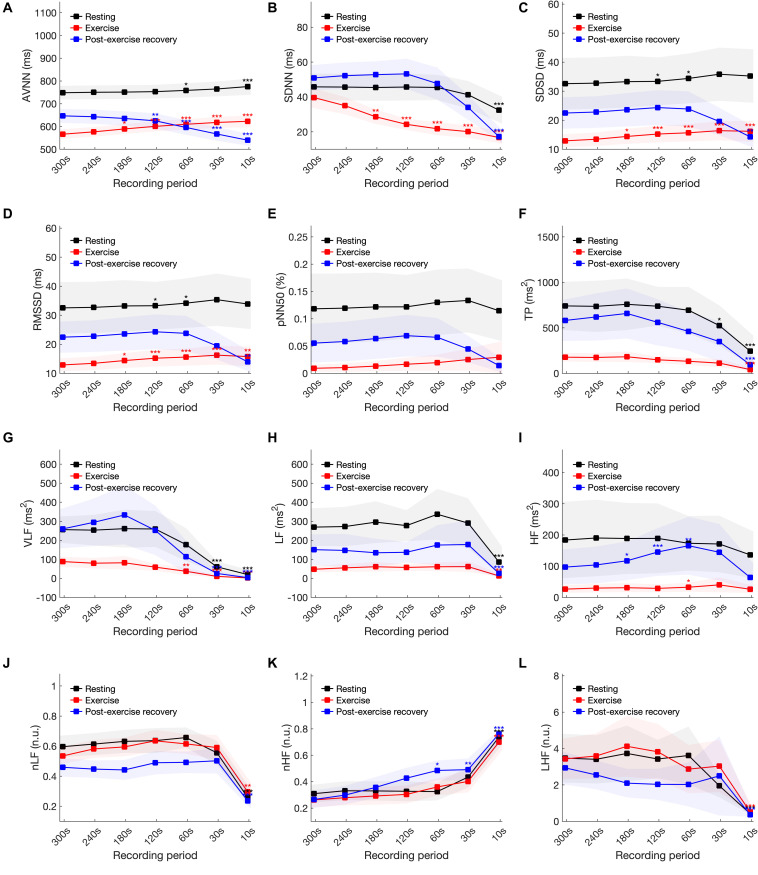
Statistical analysis of heart rate variability variables in resting, exercising, and post-exercise recovery conditions according to shortening of the analysis interval. **(A)** AVNN, **(B)** SDNN, **(C)** SDSD, **(D)** RMSSD, **(E)** pNN50, **(F)** TP, **(G)** VLF, **(H)** LF, **(I)** HF, **(J)** LH/HF, **(K)** nLF, and **(L)** nHF. Statistical significance was evaluated compared to the 300-s analysis results at *P < 0.05, **P < 0.01 and ***P < 0.001.

Figure 4 and Table 4 show the ultra- and short-term HRV results in resting, exercising, and post-exercise recovery conditions. The results of the null hypothesis test showed that all HRV variables were significantly (*P* < 0.05) different, except pNN50 in the resting and exercising conditions, and HF in the resting condition. The Bonferroni post hoc test showed significant differences (*P* < 0.05) in the resting state between ultra-short-term HRV and short-term HRV at analysis intervals of ≤10 s in the SDNN, LF, LF/HF, and HF; ≤30 s intervals in the AVNN; and ≤60 s intervals in the SDSD and RMSSD. In the exercising state, significant differences were found at ≤10-s intervals in the TP, LF, LF/HF, nLF, and nHF; at ≤60-s intervals in the VLF and HF; and at ≤180-s intervals in the SDNN, SDSD, and RMSSD. In the recovery stage, significant differences (*P* < 0.05)

**TABLE 4:** Ultra-short-term and short-term HRV group statistical results in resting, exercising, and post-exercise recovery conditions.

**HRV Variable**	**Condition**	**Analysis time**							***P*-value**
		**300 s**	**240 s**	**180 s**	**120 s**	**60 s**	**30 s**	**10 s**	
AVNN (ms)	Resting	758.0 (85.3)	759.0 (85.2)	760.0 (86.6)	762.7 (85.8)	766.5 (90.5)*	771.1 (93.5)	782.4 (100.8)***	<0.001^f^
	Exercising	571.1 (55.7)	580.9 (54.8)	593.3 (56.1)*	603.9 (58.0)***	612.3 (59.1)***	620.7 (64.3)***	624.8 (67.4)***	<0.001^f^
	Post-exercise recovery	655.3 (85.1)	651.3 (85.8)	642.4 (88.8)	631.3 (86.5)**	605.4 (78.6)***	577.7 (69.3)***	548.9 (69.3)***	<0.001^f^
SDNN (ms)	Resting	45.0 (20.7)	45.6 (20.9)	45.1 (21.1)	45.0 (21.0)	43.5 (18.8)	37.7 (20.0)	27.4 (14.6)***	<0.001^f^
	Exercising	38.9 (15.6)	34.5 (13.2)	**28.2 (10.7)**	24.3 (8.8)***	22.1 (10.2)***	20.5 (9.4)***	16.6 (8.3)***	<0.001^f^
	Post-exercise recovery	50.4 (20.4)	51.5 (20.8)	51.2 (19.8)	50.7 (21.0)	47.4 (23.5)	35.7 (9.8)	17.3 (9.8)***	<0.001^k^
SDSD (ms)	Resting	32.3 (24.1)	32.4 (23.8)	32.8 (23.9)	32.7 (21.4)*	33.0 (21.5)*	33.6 (23.3)	30.2 (17.9)	<0.01^f^
	Exercising	12.8 (5.8)	13.3 (6.0)	14.2 (6.4)*	14.8 (7.1)***	15.3 (7.8)***	16.4 (9.2)***	15.7 (9.3)***	<0.001^f^
	Post-exercise recovery	23.7 (15.9)	24.1 (16.0)	24.7 (16.9)	25.3 (17.6)	25.3 (17.4)	21.4 (13.3)	15.4 (10.7)**	<0.001^f^
RMSSD (ms)	Resting	32.2 (24.1)	32.4 (23.8)	32.8 (23.9)	32.6 (21.3)*	32.8 (21.3)*	33.1 (22.9)	29.2 (16.9)	<0.01^f^
	Exercising	12.8 (5.8)	13.2 (6.0)	14.2 (6.4)*	14.8 (7.0)***	15.2 (7.7)***	16.2 (9.0)***	15.4 (9.0)**	<0.001^f^
	Post-exercise recovery	23.7 (15.9)	24.0 (16.0)	24.7 (16.9)	25.2 (17.6)	25.2 (17.3)	21.3 (13.2)	15.0 (10.3)**	<0.001^f^
pNN50 (%)	Resting	32.2 (16.9)	32.4 (16.7)	32.8 (16.6)	32.6 (15.6)	32.8 (15.4)	33.1 (14.7)	29.2 (11.9)	0.984^k^
	Exercise	0.9 (1.7)	0.9 (2.0)	1.2 (2.0)	1.4 (2.6)	1.8 (3.8)	2.5 (5.3)	2.6 (7.4)	0.147^k^
	Post-exercise recovery	6.5 (10.6)	6.8 (10.8)	7.3 (11.1)	7.7 (11.3)	7.3 (10.3)	5.5 (7.0)	1.8 (3.8)	<0.01^k^
TP (ms^2^)	Resting	757.6 (756.2)	762.8 (813.9)	781.2 (815.6)	729.7 (553.2)	650.9 (649.0)	474.4 (619.0)*	111.2 (98.9)***	<0.001^f^
	Exercising	181.2 (123.6)	179.0 (125.8)	184.3 (137.2)	153.7 (92.5)	137.8 (113.0)	121.3 (128.9)	41.1 (48.2)***	<0.001^k^
	Post-exercise recovery	575.8 (643.4)	580.7 (706.6)	586.4 (671.2)	565.1 (738.8)	471.5 (547.9)	405.8 (547.9)	110.7 (227.6)***	<0.001^k^
VLF (ms^2^)	Resting	255.0 (188.3)	254.5 (229.5)	259.9 (292.5)	254.4 (268.1)	171.7 (237.6)	39.5 (60.3)***	5.7 (6.7)***	<0.001^k^
	Exercising	90.3 (73.5)	82.0 (80.1)	84.9 (101.3)	63.5 (53.3)	35.7 (54.3)**	9.8 (10.6)***	2.1 (2.8)***	<0.001^k^
	Post-exercise recovery	249.7 (278.0)	258.3 (301.0)	267.4 (310.8)	235.6 (368.7)	82.5 (104.4)	29.1 (104.4)***	4.6 (14.0)***	<0.001^k^
LF (ms^2^)	Resting	285.0 (292.7)	294.6 (305.3)	324.6 (323.9)	295.2 (232.2)	296.4 (240.5)	275.5 (380.6)	29.0 (31.4)***	<0.001^k^
	Exercise	47.9 (38.9)	53.4 (41.5)	55.8 (40.8)	53.5 (36.8)	62.5 (60.8)	65.0 (81.8)	11.5 (14.4)**	<0.001^k^
	Post-exercise recovery	157.8 (241.3)	153.2 (260.1)	140.4 (223.3)	140.0 (154.7)	186.3 (303.4)	200.8 (303.4)	30.5 (83.4)***	<0.001^k^
HF (ms^2^)	Resting	185.1 (335.9)	192.2 (334.9)	183.8 (290.4)	168.7 (212.2)	177.4 (240.7)	157.5 (247.7)	76.2 (67.3)	0.583^f^
	Exercising	30.9 (35.5)	34.7 (40.2)	35.7 (42.1)	33.0 (36.7)	37.7 (41.9)*	46.2 (71.2)	27.3 (34.1)	<0.05^f^
	Post-exercise recovery	113.5 (167.9)	119.7 (176.8)	132.6 (185.1)*	166.5 (228.0)***	198.4 (273.9)**	174.7 (269.0)	75.4 (132.9)	<0.001^f^
LF/HF (n.u.)	Resting	2.9 (2.5)	2.9 (2.7)	3.6 (4.1)	3.1 (2.5)	2.8 (2.4)	1.7 (1.4)	0.4 (0.3)***	<0.001^k^
	Exercising	3.0 (2.6)	3.2 (3.1)	3.8 (4.5)	3.5 (4.4)	2.6 (3.7)	2.5 (2.8)	0.5 (0.4)**	<0.001^k^
	Post-exercise recovery	2.7 (2.2)	2.4 (2.2)	2.0 (2.3)	1.8 (2.3)	1.8 (3.5)	1.3 (1.2)	0.3 (0.2)***	<0.001^k^
nLF (ms^2^)	Resting	0.6 (0.2)	0.6 (0.2)	0.6 (0.2)	0.6 (0.2)	0.6 (0.2)	0.5 (0.2)	0.2 (0.1)***	<0.001^k^
	Exercising	0.5 (0.2)	0.6 (0.2)	0.6 (0.2)	0.6 (0.2)	0.6 (0.2)	0.6 (0.2)	0.3 (0.2)***	<0.001^k^
	Post-exercise recovery	0.5 (0.2)	0.5 (0.2)	0.4 (0.2)	0.5 (0.2)	0.5 (0.2)*	0.5 (0.2)**	0.2 (0.2)***	<0.001^k^
nHF (ms^2^)	Resting	0.3 (0.2)	0.3 (0.2)	0.3 (0.2)	0.3 (0.2)	0.3 (0.2)	0.5 (0.2)	0.7 (0.1)***	<0.001^f^
	Exercising	0.3 (0.2)	0.3 (0.2)	0.3 (0.2)	0.3 (0.2)	0.4 (0.2)	0.4 (0.2)	0.7 (0.2)***	<0.001^f^
	Post-exercise recovery	0.3 (0.2)	0.3 (0.2)	0.4 (0.2)	0.5 (0.2)	0.5 (0.2)	0.5 (0.2)	0.8 (0.1)***	0.001^f^

All values are mean (SD), except for *P*-value. NN, normal-to-normal interval; AVNN, average of NN; SDNN, standard deviation of NN; SDSD, standard deviation of successive difference of NN; RMSSD, root-mean-square of successive difference of NN; pNN50, percentage of adjacent NNs that differ from each other by more than 50 ms; TP, total power; VLF, very-low-frequency power of 0.0033–0.04 Hz; LF, low-frequency power of 0.04–0.15 Hz; HF, high-frequency power of 0.15–0.4 Hz; LF/HF, ratio of low-frequency power to high-frequency power; nLF, normalized low-frequency power [LF/(TP–VLF)]; nHF, normalized high-frequency power [HF/(TP–VLF)]. Bold means statistically different cases. Statistical significance was evaluated compared to the 300-s analysis results at * *P* < 0.05, ** *P* < 0.01, *** *P* < 0.001. ^*k*^Kruskal-Wallis test. ^*f*^Friedman’s test. Bonferroni’s *post hoc* test was performed with the Kruskal-Wallis test or the Friedman test.

were found at ≤10-s intervals in the SDNN, SDSD, RMSSD, TP, LF, LF/HF, and nHF; at ≤30-s intervals in the VLF; at ≤60-s intervals in the nLF; ≤120-s intervals in the AVNN; and ≤180-s intervals in the HF.

### Cohen’s *d* Statistics

**FIGURE 5:**
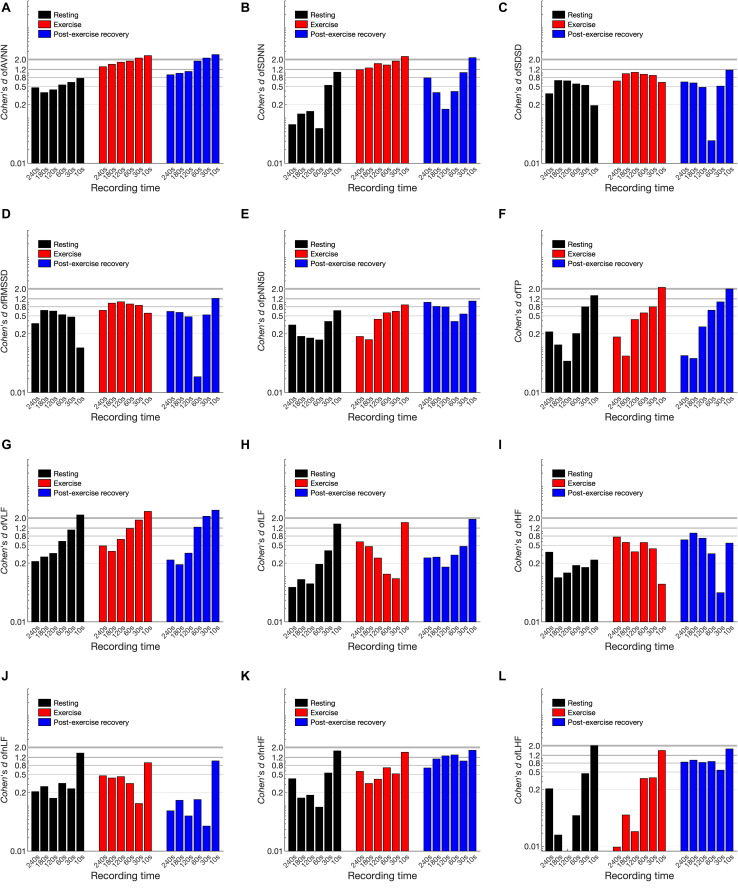
Cohen’s *d* statistics of heart rate variability variables in resting, exercising, and post-exercise recovery conditions according to shortening of the analysis interval. **(A)** AVNN, **(B)** SDNN, **(C)** SDSD, **(D)** RMSSD, **(E)** pNN50, **(F)** TP, **(G)** VLF, **(H)** LF, **(I)** HF, **(J)** LH/HF, **(K)** nLF, and **(L)** nHF.

Figure 5 and Supplementary Table 1 of the Supplementary Information (SI) show Cohen’s *d* statistics for the ultra-short-term HRV values. The minimum recording time required for each variable except for the ultra-short-term HRV values indicated as not available (n/a) was as follows for resting, exercising, and post-exercise recovery, respectively: AVNN: 120 s, n/a, and n/a; SDNN: 60 s, n/a, and n/a; SDSD: 240 s, n/a, and n/a; RMSSD: 240 s, n/a, and n/a; pNN50: 30, 120 s, and n/a; TP: 60, 120, and 120 s; VLF: 120, 180, and 120 s; LF: 30 s, n/a, and 30 s; HF: 10 s, n/a, and n/a; LF/HF: 30, 30, and 30 s; nLF: 60 s, n/a, n/a; and nHF: 30, 30 s, and n/a. The 95% confidence interval of *d* was >0.5 in all cases, and the interval was wider in the post-exercise recovery and exercising states than in the resting condition. Cohen’s *d* tended to increase with decreasing analysis interval, regardless of the experimental conditions. In addition, except for some cases, *d* was increased in the exercising and recovery conditions compared to the resting state. Based on the 240 s results, in some cases, *d* was smaller compared to the resting condition results. For example, TP was lower in the resting condition than in the exercising and post-exercise recovery conditions. In LF/HF, *d* decreased in the post-exercise recovery condition, whereas pNN50 and nHF decreased in the exercising condition.

### Pearson’s Correlation

**FIGURE 6:**
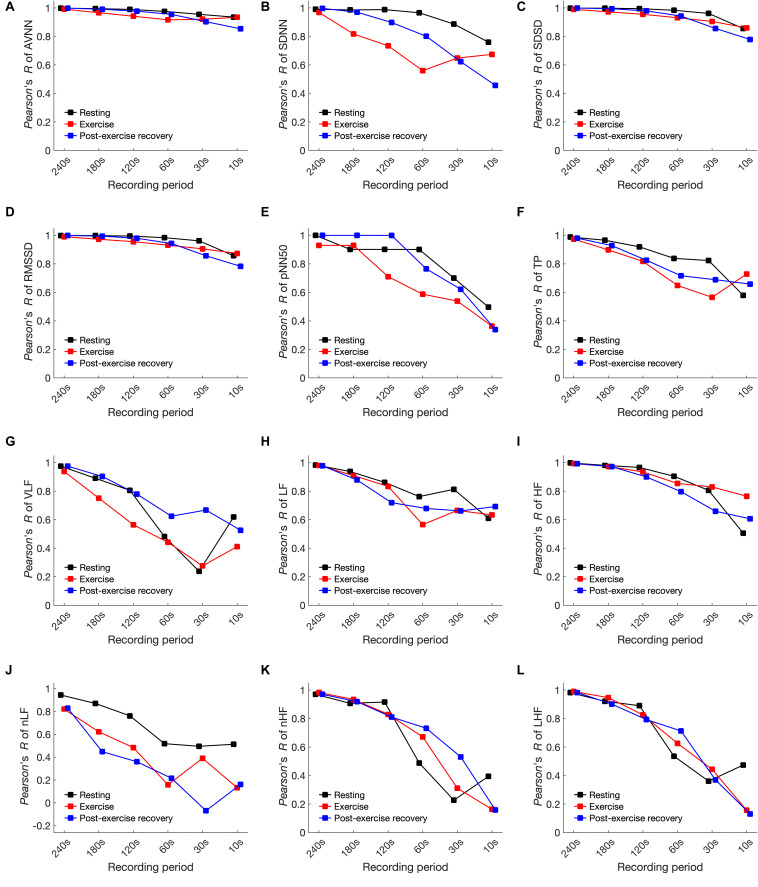
Pearson’s correlation coefficient of heart rate variability variables in resting, exercising, and post-exercise recovery conditions according to shortening of the analysis interval. **(A)** AVNN, **(B)** SDNN, **(C)** SDSD, **(D)** RMSSD, **(E)** pNN50, **(F)** TP, **(G)** VLF, **(H)** LF, **(I)** HF, **(J)** LH/HF, **(K)** nLF, and **(L)** nHF.

Figure 6 and Supplementary Table 2 of the SI show the results of Pearson’s correlation analysis. In all cases, the correlation coefficient decreased according to the shortening of the analysis interval. The trend of the decrease was different for each condition, but variables such as SDNN, pNN50, TP, VLF, and LF/HF showed clear differences in each condition. In most cases, the correlation coefficient was largely decreased in the dynamic condition compared to the resting condition as the analysis interval decreased. The minimum analysis interval required for each HRV variable for resting, exercising, and post-exercise recovery, respectively, was as follows: AVNN: 10, 10, and 10 s; SDNN: 30, 180, and 60 s; SDSD: 10, 10, and 30 s; RMSSD: 10, 10, and 30 s; pNN50: 60, 180, and 120 s; TP: 30, 120, and 120 s; VLF: 120, 240, and 180 s; LF: 120, 120, and 180 s; HF: 30, 30, and 120 s; LF/HF: 180, 240, and 240 s; nLF: 120, 120, and 120 s; and nHF: 120, 120, and 180 s. The correlation coefficient also showed a drastic decrease in specific analysis intervals. For example, the correlation coefficient in the resting pNN50 decreased from 0.700 to 0.497 at <30-s intervals, the VLF from 0.806 to 0.482 at <120-s intervals, and 0.916–0.488 at <120-s intervals.

### Bland–Altman Limits of Agreement

**FIGURE 7:**
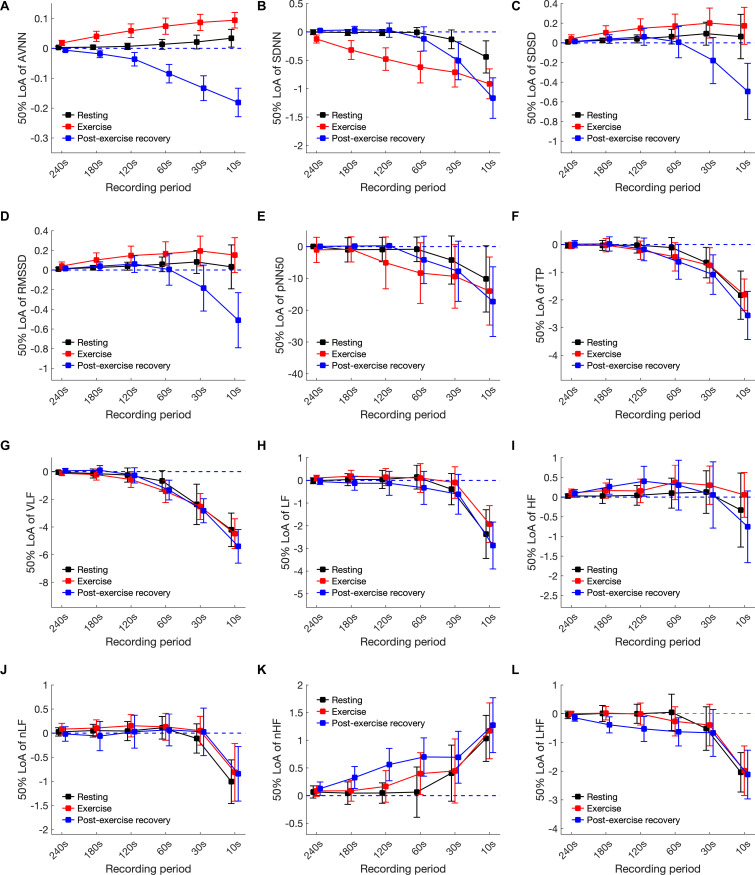
The 50% limits of agreement of heart rate variability variables in resting, exercising, and post-exercise recovery conditions according to shortening of the analysis interval. **(A)** AVNN, **(B)** SDNN, **(C)** SDSD, **(D)** RMSSD, **(E)** pNN50, **(F)** TP, **(G)** VLF, **(H)** LF, **(I)** HF, **(J)** LH/HF, **(K)** nLF, and **(L)** nHF.

Figure 7 and Supplementary Table 3 in the SI, show increases in bias, and the width of the 50% LoA interval was observed to increase as the analysis interval decreased for all HRV variables in every condition. This means that the shorter the analysis interval, the greater the likelihood of error. The minimum time for ultra-short-term HRV analysis based on 50% LoA for resting, exercising, and post-exercise recovery states, respectively, was as follows: AVNN: 30 s, n/a, and n/a; SDNN: 30 s, n/a, and n/a; SDSD: 240, 240, and 30 s; RMSSD: 10, 240, and 30 s; pNN50: 10, 30 s, and n/a; TP: 60, 60, and 60 s; VLF: 60, 120, and 120 s; LF: 30, 30, and 30 s; HF:10 s, n/a, and 240 s; LF/HF: 30, 30, and 30 s; nLF: 30, 120 s, and n/a; and nHF: 30, 30 s, and n/a.

## Discussion

Unlike the existing ultra-short-term HRV studies conducted in resting conditions, we focused on analyzing the minimum time required for ultra-short-term HRV analysis in resting, exercising, and post-exercise recovery conditions. In this study, we used statistical methods such as null hypothesis testing, Cohen’s *d* statistics, Pearson’s correlation, or Bland-Altman LoA in combination to derive the required time for ultra-short-term HRV analysis.

### The Minimum Time Requirement for Ultra-Short-Term HRV Analysis

Table 5 shows the summary of the minimum time required for the analysis of ultra-short-term HRV variables in each condition investigated using various statistical metrics. Although the minimum requirement times derived from the various analysis methods differed from each other, in general, the minimum analysis time requirement in the dynamic condition increased in all results. In addition, while Cohen’s *d*, the LoA, and the null hypothesis tests showed a relatively longer minimum time requirement in the time domain analysis and a shorter minimum time requirement in the frequency domain analysis, Pearson’s *R* tests showed a short minimum time requirement for time-domain analysis and a longer time requirement for frequency domain analysis, revealing mutually opposite results. Although it is very difficult to synthesize these results, conclusions can be drawn from the most strict or lenient conditions that satisfy all conditions. Strict conditions are mainly derived from the results of Cohen’s *d*, the LoA, or the null hypothesis test, indicating that many HRV variables cannot be used for dynamic analysis or require at least 120 s of analysis time for some variables. Lenient conditions showed that the time domain variables were mainly derived from Cohen’s *d*, the LoA, and the hypothesis tests, while the frequency domain variables were mainly derived from Pearson’s *R*, which showed the ability of ultra-short-term HRV analysis in 60-s recording intervals. While the two suggestions show a difference in the minimum required time, they suggest that in common, longer recording is required in almost all cases for the ultra-short-term HRV analysis of dynamic conditions compared to the static condition.

**TABLE 5:** Suggested minimum analysis intervals for ultra-short-term heart rate variability analysis according to the statistical metrics.

**Metric**	**Condition**	**HRV Variables (s)**											
		**AVNN**	**SDNN**	**SDSD**	**RMSSD**	**pNN50**	**TP**	**VLF**	**LF**	**HF**	**LF/HF**	**nLF**	**nHF**
Cohen’s *d*	Resting	120	60	240	240	30	60	120	30	10	30	60	30
	Exercising	n/a	n/a	n/a	n/a	120	120	180	n/a	n/a	30	n/a	30
	Post-exercise recovery	n/a	n/a	n/a	n/a	n/a	120	120	30	n/a	30	n/a	n/a
Pearson’s *R*	Resting	10	30	10	10	60	30	120	120	30	180	120	120
	Exercising	10	180	10	10	180	120	240	120	30	240	120	120
	Post-exercise recovery	10	60	30	30	120	120	180	180	120	240	120	180
Limits of agreements	Resting	30	30	240	10	10	60	60	30	10	30	30	30
	Exercising	n/a	n/a	240	240	30	60	120	30	n/a	30	120	30
	Post-exercise recovery	n/a	n/a	30	30	n/a	60	120	30	240	30	n/a	n/a
Hypothesis test	Resting	120	30	180	180	10	60	60	30	10	30	30	30
	Exercising	240	240	240	240	10	30	120	30	120	30	30	30
	Post-exercise recovery	180	30	30	30	10	30	60	30	240	30	120	30
Recommendation (strict)	Resting	120	60	240	240	60	60	120	120	30	180	120	120
	Exercising	240	n/a	n/a	n/a	180	120	240	n/a	n/a	240	n/a	120
	Post-exercise recovery	180	n/a	n/a	n/a	n/a	120	180	180	n/a	240	n/a	n/a
Recommendation (lenient)	Resting	10	30	10	10	10	60	60	30	10	30	30	30
	Exercising	10	180	10	10	10	30	120	30	30	30	30	30
	Post-exercise recovery	10	60	30	30	10	30	60	30	120	30	120	30

*Decision criteria for availability: *d* < 0.5 (small) for Cohen’s *d*, *R* > 0.8 for Pearson’s *R*, 50% limits of agreement, *P* < 0.05 for the null hypothesis test. n/a, not available. The strict condition was a combination of the values with the longest analysis intervals in the same conditions and the lenient condition was a combination of the shortest analysis intervals in the same conditions.*

### Ultra-Short-Term HRV in Dynamic Conditions

#### HRV in Exercise Conditions

Before discussing ultra-short-term HRV in dynamic conditions, consideration should be given to cardiac autonomic regulation during exercising or post-exercise recovery. In the emerged cardiac autonomic regulation model (Raven et al., 2006; Nobrega et al., 2014; White and Raven, 2014; Michelini et al., 2015), the HR rapidly increases, primarily mediated by reduced cardiac parasympathetic neural activity and reductions in cardiac sympathetic neural activity. In cardiac rhythm regulation, both the SNS (Sympathetic Nervous System) and PNS (Parasympathetic Nervous System) regulate HR throughout the entire exercise duration, where the SNS works as a tone-setter and the PNS operates as a rapid responder/modulator (Michael et al., 2017). In resting or low-level activity conditions, parasympathetic control is dominant. However, sympathetic control becomes dominant according to increases in exercise intensity (White and Raven, 2014). As a result, higher exercise intensity was reportedly associated with decreases in the SDNN (Tulppo et al., 1996; Hautala et al., 2003); RMSSD (Tulppo et al., 1996; Leicht et al., 2008; Boettger et al., 2010; Karapetian et al., 2012); LF, HF, and TP (Perini et al., 1989; Tulppo et al., 1996; Avery et al., 2001; Hautala et al., 2003; Povea et al., 2005; Casties et al., 2006; Spadacini et al., 2006; Fisher et al., 2009; Boettger et al., 2010; Karapetian et al., 2012); and HF (Cottin et al., 2006, 2007). Typically, the nLF increases during low-moderate intensity exercise and decreases during higher intensity exercise, whereas the nHF shows the opposite response (Perini et al., 1990, 1993; Hautala et al., 2003; Pichon et al., 2004; Povea et al., 2005; Martinmäki and Rusko, 2008), although conflicting responses have also been reported (Avery et al., 2001; Casties et al., 2006). The LF/HF demonstrates inconsistent responses to exercise. Some studies reported an increase in low-moderate intensity exercise and a decrease during higher intensity exercises (Radaelli et al., 1996; Tulppo et al., 1996; Hautala et al., 2003). However, other studies reported a progressive decrease from rest with increasing exercise intensity (Casties et al., 2006) or a progressive increase from rest (Saito and Nakamura, 1995; Avery et al., 2001).

#### HRV in Post-exercise Recovery Conditions

In post-exercise recovery conditions, the aforementioned processes mediating cardio-acceleration during exercise occur in reverse, and finally, the HR and HRV demonstrate a time-dependent recovery and eventual return to pre-exercise levels (Stanley et al., 2013). Heart rate changes in the post-exercise recovery period are not theoretically well-established, and there is a view that rapid HR reduction immediately after exercise is influenced by parasympathetic reactions (Cole et al., 1999; Coote, 2010; Peçanha et al., 2014) or is affected by sympathetic activity as well (Kannankeril et al., 2004; Pichon et al., 2004). All of the above demonstrate that during exercise or recovery, either the SNS or the PNS may be dominant and that this effect may vary from person to person. In addition, the balance of the SNS and PNS may change according to exercise intensity and the post-exercise recovery status. For example, which nervous system will be dominant depends upon the exercise intensity, with the PNS dominant in low-moderate exercise and the SNS in strenuous exercise (White and Raven, 2014). Similarly, during recovery, autonomic activity may be different depending upon early and late recovery periods. In the early phase of recovery, although some evidence has suggested sympathetic involvement as well (Nandi and Spodick, 1977; Kannankeril et al., 2004; Pichon et al., 2004), the “fast phase” of HR recovery has often been attributed to parasympathetic reactivation (Perini et al., 1989; Imai et al., 1994; Cole et al., 1999; Peçanha et al., 2014), and in the late phase of recovery, a more gradual “slow phase” of cardio-deceleration is observed, mediated by both progressive parasympathetic reactivation and sympathetic withdrawal (Michael et al., 2017).

Thus, in a dynamic state, such as movement or recovery, the response of the autonomic nervous system is not steady-state, but transient. Transient indicates a status in which the autonomic nervous system is not stabilized and continuously changes. Therefore, longer analysis intervals than transient intervals can be used to ensure stable analysis without significant differences compared to the steady-state. Therefore, finding the minimum required interval for ultra-short-term HRV means finding the transient interval under each condition? Thus, it is generally expected that the time required for HRV analysis in the dynamic condition will be longer than in the resting condition, assuming steady-state. In addition, since the length of the transient interval for exercise and recovery differs for each individual, it is necessary to consider the time required for ultra-short-term HRV analysis under the various conditions.

### Limitations

This study was the first study to investigate the application of ultra-short-term HRV in dynamic conditions. However, the following limitations impact the generalizability of the results and emphasize the necessity of further research. First, the conventional HF band of 0.15–0.40 Hz was used in this research. However, this band may not be suitable during exercise where higher respiratory frequencies are observed. This was already suggested by the results of previous studies where the standard spectral HRV analysis was more susceptible to anomalies compared to the time domain analysis (Ng et al., 2009), and thus, should not be used in exercise conditions (Michael et al., 2017).

Second, because the subjects of this study were limited to young and healthy adults, the results of this study cannot be generalized to all age groups, including diseased populations, and the number of subjects was also insufficient for parameter validation. In addition, this study did not include the analysis of various activities, such as walking, running, sitting, standing, and climbing stairs that can be encountered in daily life nor did it include an analysis by emotional state. Therefore, for the application to general mobile healthcare research, expansion of the research to various dynamic states is required.

## Conclusion

Dynamic behavior causes non-stationary transient changes in the autonomic nervous system, which can greatly affect the minimum interval required for HRV analysis. The results of this study suggest that the application of ultra-short-term HRV in dynamic conditions, such as exercising and post-exercise recovery periods that may occur in daily life, requires dedicated criteria for the analysis interval that is different from the existing resting ultra-short-term HRV analysis criteria. In conclusion, ultra-short-term HRV analysis in dynamic conditions required longer analysis intervals compared to resting conditions, and in dynamic conditions, when strict criteria are applied to satisfy various statistical analysis techniques, ultra-short-term HRV analysis is not recommended.

## Data Availability Statement

The data analyzed in this study is subject to the following licenses/restrictions: The datasets analyzed during the current study are not publicly available due to privacy issue but are available from the corresponding author on reasonable request. Requests to access these datasets should be directed to HS, hangsik.shin@jnu.ac.kr.

## Ethics Statement

The studies involving human participants were reviewed and approved by Asan medical center. The patients/participants provided their written informed consent to participate in this study.

## Author Contributions

HS and JK designed the experimental protocol. JK carried out experiment and collected data. HS and HSS analyzed data statistically. HS supervised the project. All authors contributed to the article and approved the submitted version.

## Conflict of Interest

The authors declare that the research was conducted in the absence of any commercial or financial relationships that could be construed as a potential conflict of interest.
