# Supplementary material for: Is Ultra-Short-Term Heart Rate Variability Valid in Non-static Conditions?
Source: Front Physiol. 2021 Mar 30;12:596060. doi: 10.3389/fphys.2021.596060 (PMC8042416; doi:10.3389/fphys.2021.596060)
Supplement: Supplementary file 1 [file Table_1.DOCX]

**Supplementary Information**

Table S1. Cohen’s *d* statistics for ultra-short-term heart rate variability variables in each condition.

| HRV variable | Condition | Mean (95 % of confidence interval) | | | | | | |
| --- | --- | --- | --- | --- | --- | --- | --- | --- |
|  |  | 300 s | 240 s | 180 s | 120 s | 60 s | 30 s | 10 s |
| AVNN (ms) | Resting | 0.47 (0.00-1.00) | 0.37 (0.00-0.90) | 0.42 (0.00-0.95) | 0.55 (0.01-1.08) | 0.62 (0.09-1.16) | 0.77 (0.22-1.31) | 0.47 (0.00-1.00) |
|  | Exercise | 1.38 (0.80-1.96) | 1.57 (0.97-2.16) | 1.75 (1.13-2.36) | 1.84 (1.22-2.47) | 2.16 (1.50-2.82) | 2.44 (1.75-3.13) | 1.38 (0.80-1.96) |
|  | Post-exercise recovery | 0.92 (0.36-1.47) | 0.98 (0.43-1.54) | 1.09 (0.53-1.65) | 1.84 (1.22-2.47) | 2.16 (1.50-2.82) | 2.56 (1.85-3.26) | 0.92 (0.36-1.47) |
| SDNN (ms) | Resting | 0.07 (0.00-0.60) | 0.12 (0.00-0.65) | 0.14 (0.00-0.67) | 0.06 (0.00-0.58) | 0.54 (0.00-1.07) | 1.05 (0.49-1.60) | 0.07 (0.00-0.60) |
|  | Exercise | 1.18 (0.62-1.75) | 1.3 (0.72-1.88) | 1.63 (1.03-2.24) | 1.51 (0.91-2.10) | 1.84 (1.21-2.46) | 2.33 (1.66-3.01) | 1.18 (0.62-1.75) |
|  | Post-exercise recovery | 0.78 (0.24-1.32) | 0.37 (0.00-0.90) | 0.16 (0.00-0.68) | 0.39 (0.00-0.92) | 1.02 (0.47-1.58) | 2.2 (1.54-2.86) | 0.78 (0.24-1.32) |
| SDSD (ms) | Resting | 0.35 (0.00-0.88) | 0.68 (0.15-1.22) | 0.67 (0.13-1.21) | 0.57 (0.04-1.11) | 0.54 (0.00-1.07) | 0.19 (0.00-0.72) | 0.35 (0.00-0.88) |
|  | Exercise | 0.67 (0.13-1.21) | 0.97 (0.42-1.52) | 1.04 (0.48-1.60) | 0.94 (0.39-1.49) | 0.89 (0.34-1.44) | 0.62 (0.08-1.16) | 0.67 (0.13-1.21) |
|  | Post-exercise recovery | 0.64 (0.10-1.17) | 0.6 (0.07-1.14) | 0.48 (0.00-1.01) | 0.03 (0.00-0.56) | 0.51 (0.00-1.05) | 1.16 (0.60-1.73) | 0.64 (0.10-1.17) |
| RMSSD (ms) | Resting | 0.34 (0.00-0.87) | 0.66 (0.12-1.20) | 0.64 (0.10-1.18) | 0.53 (0.00-1.06) | 0.47 (0.00-1.00) | 0.1 (0.00-0.62) | 0.34 (0.00-0.87) |
|  | Exercise | 0.66 (0.13-1.20) | 0.96 (0.41-1.52) | 1.03 (0.47-1.59) | 0.92 (0.37-1.47) | 0.86 (0.31-1.41) | 0.58 (0.04-1.11) | 0.66 (0.13-1.20) |
|  | Post-exercise recovery | 0.63 (0.09-1.17) | 0.6 (0.06-1.13) | 0.48 (0.00-1.01) | 0.02 (0.00-0.55) | 0.53 (0.00-1.06) | 1.23 (0.66-1.80) | 0.63 (0.09-1.17) |
| pNN50 (%) | Resting | 0.31 (0.00-0.84) | 0.18 (0.00-0.70) | 0.16 (0.00-0.69) | 0.15 (0.00-0.67) | 0.38 (0.00-0.91) | 0.66 (0.12-1.19) | 0.31 (0.00-0.84) |
|  | Exercise | 0.18 (0.00-0.70) | 0.15 (0.00-0.67) | 0.42 (0.00-0.95) | 0.59 (0.05-1.12) | 0.63 (0.10-1.17) | 0.88 (0.33-1.43) | 0.18 (0.00-0.70) |
|  | Post-exercise recovery | 1.01 (0.45-1.56) | 0.81 (0.27-1.36) | 0.79 (0.25-1.33) | 0.38 (0.00-0.91) | 0.55 (0.02-1.09) | 1.07 (0.51-1.63) | 1.01 (0.45-1.56) |
| TP (ms^2^) | Resting | 0.22 (0.00-0.75) | 0.11 (0.00-0.64) | 0.05 (0.00-0.57) | 0.2 (0.00-0.73) | 0.8 (0.25-1.34) | 1.42 (0.83-2.00) | 0.22 (0.00-0.75) |
|  | Exercise | 0.17 (0.00-0.70) | 0.06 (0.00-0.59) | 0.42 (0.00-0.95) | 0.58 (0.05-1.12) | 0.8 (0.25-1.34) | 2.14 (1.49-2.80) | 0.17 (0.00-0.70) |
|  | Post-exercise recovery | 0.07 (0.00-0.59) | 0.06 (0.00-0.58) | 0.29 (0.00-0.81) | 0.67 (0.13-1.21) | 1.03 (0.47-1.58) | 1.98 (1.34-2.62) | 0.07 (0.00-0.59) |
| VLF (ms^2^) | Resting | 0.22 (0.00-0.74) | 0.27 (0.00-0.80) | 0.33 (0.00-0.86) | 0.61 (0.07-1.15) | 1.09 (0.53-1.65) | 2.35 (1.67-3.03) | 0.22 (0.00-0.74) |
|  | Exercise | 0.48 (0.00-1.01) | 0.37 (0.00-0.89) | 0.67 (0.13-1.21) | 1.18 (0.61-1.75) | 1.81 (1.19-2.43) | 2.81 (2.07-3.55) | 0.48 (0.00-1.01) |
|  | Post-exercise recovery | 0.24 (0.00-0.76) | 0.19 (0.00-0.71) | 0.33 (0.00-0.86) | 1.26 (0.68-1.83) | 2.19 (1.53-2.85) | 2.99 (2.23-3.75) | 0.24 (0.00-0.76) |
| LF (ms^2^) | Resting | 0.06 (0.00-0.58) | 0.09 (0.00-0.61) | 0.07 (0.00-0.59) | 0.19 (0.00-0.71) | 0.38 (0.00-0.91) | 1.49 (0.89-2.08) | 0.06 (0.00-0.58) |
|  | Exercise | 0.59 (0.06-1.13) | 0.47 (0.00-1.00) | 0.26 (0.00-0.79) | 0.11 (0.00-0.64) | 0.09 (0.00-0.61) | 1.59 (0.99-2.20) | 0.59 (0.06-1.13) |
|  | Post-exercise recovery | 0.26 (0.00-0.79) | 0.27 (0.00-0.80) | 0.16 (0.00-0.69) | 0.30 (0.00-0.83) | 0.47 (0.00-1.00) | 1.87 (1.25-2.50) | 0.26 (0.00-0.79) |
| HF (ms^2^) | Resting | 0.35 (0.00-0.88) | 0.1 (0.00-0.62) | 0.12 (0.00-0.65) | 0.18 (0.00-0.70) | 0.16 (0.00-0.69) | 0.24 (0.00-0.76) | 0.35 (0.00-0.88) |
|  | Exercise | 0.76 (0.22-1.30) | 0.58 (0.04-1.11) | 0.36 (0.00-0.88) | 0.57 (0.04-1.11) | 0.42 (0.00-0.95) | 0.07 (0.00-0.59) | 0.76 (0.22-1.30) |
|  | Post-exercise recovery | 0.66 (0.12-1.20) | 0.93 (0.38-1.48) | 0.71 (0.17-1.25) | 0.32 (0.00-0.85) | 0.04 (0.00-0.57) | 0.56 (0.02-1.09) | 0.66 (0.12-1.20) |
| LF/HF (n.u.) | Resting | 0.21 (0.00-0.73) | 0.27 (0.00-0.80) | 0.15 (0.00-0.67) | 0.32 (0.00-0.85) | 0.24 (0.00-0.77) | 1.5 (0.91-2.09) | 0.21 (0.00-0.73) |
|  | Exercise | 0.47 (0.00-1.00) | 0.42 (0.00-0.95) | 0.45 (0.00-0.98) | 0.32 (0.00-0.84) | 0.11 (0.00-0.64) | 0.92 (0.37-1.47) | 0.47 (0.00-1.00) |
|  | Post-exercise recovery | 0.08 (0.00-0.60) | 0.13 (0.00-0.66) | 0.06 (0.00-0.58) | 0.14 (0.00-0.66) | 0.04 (0.00-0.56) | 1 (0.45-1.56) | 0.08 (0.00-0.60) |
| nLF (ms^2^) | Resting | 0.40 (0.00-0.93) | 0.15 (0.00-0.67) | 0.17 (0.00-0.70) | 0.09 (0.00-0.62) | 0.54 (0.01-1.08) | 1.67 (1.06-2.28) | 0.4 (0.00-0.93) |
|  | Exercise | 0.59 (0.05-1.12) | 0.32 (0.00-0.84) | 0.39 (0.00-0.92) | 0.71 (0.17-1.25) | 0.52 (0.00-1.05) | 1.56 (0.96-2.16) | 0.59 (0.05-1.12) |
|  | Post-exercise recovery | 0.70 (0.16-1.24) | 1.11 (0.55-1.67) | 1.29 (0.72-1.87) | 1.36 (0.78-1.94) | 1 (0.44-1.55) | 1.73 (1.12-2.35) | 0.7 (0.16-1.24) |
| nHF (ms^2^) | Resting | 0.21 (0.00-0.73) | 0.02 (0.00-0.54) | 0.01 (0.00-0.53) | 0.05 (0.00-0.57) | 0.46 (0.00-0.99) | 2.01 (1.37-2.65) | 0.21 (0.00-0.73) |
|  | Exercise | 0.01 (0.00-0.53) | 0.05 (0.00-0.58) | 0.02 (0.00-0.55) | 0.36 (0.00-0.88) | 0.37 (0.00-0.90) | 1.55 (0.95-2.15) | 0.01 (0.00-0.53) |
|  | Post-exercise recovery | 0.84 (0.30-1.39) | 0.93 (0.38-1.48) | 0.82 (0.28-1.37) | 0.87 (0.32-1.41) | 0.55 (0.02-1.08) | 1.68 (1.07-2.29) | 0.84 (0.30-1.39) |
| NN: normal-to-normal interval; AVNN: Average of NN; SDNN: standard deviation of NN; SDSD: standard deviation of successive difference of NN; RMSSD: root-mean-square of successive difference of NN; pNN50: percentage of adjacent NNs that differ from each other by more than 50 ms; TP: total power; VLF: very-low-frequency power of (0.0033–0.04) Hz; LF: low-frequency power of (0.04–0.15) Hz; HF: high-frequency power of (0.15–0.4) Hz; LF/HF: ratio of low-frequency power to high-frequency power; nLF: normalized low-frequency power (LF/(TP–VLF)); nHF: normalized high-frequency power (HF/(TP–VLF)). | | | | | | | | |

Table S2. Pearson’s correlation coefficient (*R*) for ultra-short-term heart rate variability variables in each condition.

| HRV variable | Condition | Mean (95 % of confidence interval) | | | | | | |
| --- | --- | --- | --- | --- | --- | --- | --- | --- |
|  |  | 300 s | 240 s | 180 s | 120 s | 60 s | 30 s | 10 s |
| AVNN (ms) | Resting | 1.00 (1.00-1.00) | 1.00 (0.99-1.00) | 0.99 (0.98-1.00) | 0.98 (0.95-0.99) | 0.96 (0.91-0.98) | 0.94 (0.86-0.97) | 1.00 (1.00-1.00) |
|  | Exercise | 0.99 (0.98-1.00) | 0.97 (0.93-0.98) | 0.94 (0.88-0.97) | 0.92 (0.83-0.96) | 0.92 (0.83-0.96) | 0.93 (0.86-0.97) | 0.99 (0.98-1.00) |
|  | Post-exercise recovery | 1.00 (1.00-1.00) | 0.99 (0.98-1.00) | 0.98 (0.95-0.99) | 0.96 (0.90-0.98) | 0.9 (0.80-0.95) | 0.85 (0.71-0.93) | 1.00 (1.00-1.00) |
| SDNN (ms) | Resting | 0.99 (0.98-1.00) | 0.99 (0.97-0.99) | 0.99 (0.97-0.99) | 0.97 (0.93-0.98) | 0.89 (0.77-0.95) | 0.76 (0.54-0.88) | 0.99 (0.98-1.00) |
|  | Exercise | 0.97 (0.93-0.99) | 0.82 (0.64-0.91) | 0.73 (0.50-0.87) | 0.56 (0.24-0.77) | 0.65 (0.36-0.82) | 0.67 (0.40-0.84) | 0.97 (0.93-0.99) |
|  | Post-exercise recovery | 1.00 (0.99-1.00) | 0.97 (0.94-0.99) | 0.90 (0.79-0.95) | 0.8 (0.61-0.90) | 0.62 (0.33-0.81) | 0.46 (0.10-0.71) | 1.00 (0.99-1.00) |
| SDSD (ms) | Resting | 1.00 (1.00-1.00) | 1.00 (1.00-1.00) | 1.00 (0.99-1.00) | 0.98 (0.96-0.99) | 0.96 (0.92-0.98) | 0.86 (0.71-0.93) | 1.00 (1.00-1.00) |
|  | Exercise | 0.99 (0.98-1.00) | 0.97 (0.94-0.99) | 0.96 (0.91-0.98) | 0.93 (0.86-0.97) | 0.91 (0.80-0.96) | 0.86 (0.72-0.93) | 0.99 (0.98-1.00) |
|  | Post-exercise recovery | 1.00 (1.00-1.00) | 0.99 (0.99-1.00) | 0.98 (0.96-0.99) | 0.94 (0.88-0.97) | 0.86 (0.71-0.93) | 0.78 (0.57-0.89) | 1.00 (1.00-1.00) |
| RMSSD (ms) | Resting | 1.00 (1.00-1.00) | 1.00 (1.00-1.00) | 1.00 (0.99-1.00) | 0.98 (0.96-0.99) | 0.96 (0.92-0.98) | 0.86 (0.71-0.93) | 1.00 (1.00-1.00) |
|  | Exercise | 0.99 (0.98-1.00) | 0.97 (0.94-0.99) | 0.96 (0.91-0.98) | 0.93 (0.86-0.97) | 0.91 (0.80-0.96) | 0.87 (0.74-0.94) | 0.99 (0.98-1.00) |
|  | Post-exercise recovery | 1.00 (1.00-1.00) | 0.99 (0.99-1.00) | 0.98 (0.96-0.99) | 0.94 (0.88-0.97) | 0.86 (0.71-0.93) | 0.78 (0.58-0.89) | 1.00 (1.00-1.00) |
| pNN50 (%) | Resting | 1.00 (1.00-1.00) | 0.90 (0.80-0.95) | 0.90 (0.80-0.95) | 0.90 (0.80-0.95) | 0.70 (0.44-0.85) | 0.50 (0.15-0.73) | 1.00 (1.00-1.00) |
|  | Exercise | 0.93 (0.85-0.97) | 0.93 (0.85-0.97) | 0.71 (0.46-0.86) | 0.59 (0.27-0.79) | 0.54 (0.21-0.76) | 0.36 (0.00-0.65) | 0.93 (0.85-0.97) |
|  | Post-exercise recovery | 1.00 (1.00-1.00) | 1.00 (1.00-1.00) | 1.00 (1.00-1.00) | 0.77 (0.55-0.89) | 0.62 (0.32-0.81) | 0.34 (0.00-0.63) | 1.00 (1.00-1.00) |
| TP (ms^2^) | Resting | 0.99 (0.97-0.99) | 0.97 (0.93-0.98) | 0.92 (0.83-0.96) | 0.84 (0.68-0.92) | 0.82 (0.65-0.92) | 0.58 (0.26-0.78) | 0.99 (0.97-0.99) |
|  | Exercise | 0.97 (0.94-0.99) | 0.9 (0.79-0.95) | 0.82 (0.64-0.91) | 0.65 (0.36-0.82) | 0.57 (0.24-0.77) | 0.73 (0.49-0.87) | 0.97 (0.94-0.99) |
|  | Post-exercise recovery | 0.98 (0.96-0.99) | 0.93 (0.85-0.97) | 0.83 (0.65-0.92) | 0.72 (0.47-0.86) | 0.69 (0.42-0.84) | 0.66 (0.38-0.83) | 0.98 (0.96-0.99) |
| VLF (ms^2^) | Resting | 0.98 (0.95-0.99) | 0.89 (0.77-0.95) | 0.81 (0.62-0.91) | 0.48 (0.13-0.72) | 0.24 (0.00-0.56) | 0.62 (0.32-0.81) | 0.98 (0.95-0.99) |
|  | Exercise | 0.94 (0.87-0.97) | 0.75 (0.52-0.88) | 0.56 (0.24-0.77) | 0.44 (0.08-0.70) | 0.28 (0.00-0.59) | 0.41 (0.05-0.68) | 0.94 (0.87-0.97) |
|  | Post-exercise recovery | 0.98 (0.95-0.99) | 0.90 (0.80-0.95) | 0.78 (0.57-0.89) | 0.62 (0.33-0.81) | 0.67 (0.39-0.83) | 0.53 (0.19-0.75) | 0.98 (0.95-0.99) |
| LF (ms^2^) | Resting | 0.98 (0.96-0.99) | 0.94 (0.87-0.97) | 0.86 (0.72-0.93) | 0.76 (0.54-0.88) | 0.81 (0.63-0.91) | 0.61 (0.31-0.80) | 0.98 (0.96-0.99) |
|  | Exercise | 0.98 (0.96-0.99) | 0.91 (0.81-0.96) | 0.83 (0.67-0.92) | 0.57 (0.24-0.78) | 0.67 (0.39-0.83) | 0.63 (0.34-0.81) | 0.98 (0.96-0.99) |
|  | Post-exercise recovery | 0.98 (0.95-0.99) | 0.88 (0.75-0.94) | 0.72 (0.47-0.86) | 0.68 (0.41-0.84) | 0.66 (0.38-0.83) | 0.69 (0.43-0.85) | 0.98 (0.95-0.99) |
| HF (ms^2^) | Resting | 1.00 (1.00-1.00) | 0.98 (0.96-0.99) | 0.97 (0.93-0.98) | 0.90 (0.80-0.96) | 0.81 (0.62-0.91) | 0.51 (0.16-0.74) | 1.00 (1.00-1.00) |
|  | Exercise | 0.99 (0.99-1.00) | 0.97 (0.94-0.99) | 0.94 (0.86-0.97) | 0.85 (0.71-0.93) | 0.83 (0.66-0.92) | 0.76 (0.55-0.89) | 0.99 (0.99-1.00) |
|  | Post-exercise recovery | 0.99 (0.98-1.00) | 0.97 (0.94-0.99) | 0.90 (0.79-0.95) | 0.80 (0.60-0.90) | 0.66 (0.38-0.83) | 0.61 (0.30-0.80) | 0.99 (0.98-1.00) |
| LF/HF (n.u.) | Resting | 0.94 (0.88-0.97) | 0.87 (0.74-0.94) | 0.76 (0.54-0.88) | 0.52 (0.18-0.75) | 0.5 (0.15-0.73) | 0.51 (0.17-0.74) | 0.94 (0.88-0.97) |
|  | Exercise | 0.82 (0.64-0.91) | 0.62 (0.32-0.81) | 0.48 (0.13-0.73) | 0.16 (0.00-0.50) | 0.39 (0.02-0.67) | 0.13 (0.00-0.48) | 0.82 (0.64-0.91) |
|  | Post-exercise recovery | 0.83 (0.66-0.92) | 0.45 (0.09-0.70) | 0.36 (0.00-0.65) | 0.22 (0.00-0.54) | -0.07 (0.00-0.31) | 0.16 (0.00-0.50) | 0.83 (0.66-0.92) |
| nLF (ms^2^) | Resting | 0.97 (0.94-0.99) | 0.91 (0.81-0.96) | 0.92 (0.82-0.96) | 0.49 (0.14-0.73) | 0.23 (0.00-0.55) | 0.39 (0.02-0.67) | 0.97 (0.94-0.99) |
|  | Exercise | 0.98 (0.96-0.99) | 0.93 (0.86-0.97) | 0.83 (0.66-0.92) | 0.67 (0.40-0.84) | 0.31 (0.00-0.61) | 0.16 (0.00-0.51) | 0.98 (0.96-0.99) |
|  | Post-exercise recovery | 0.97 (0.94-0.99) | 0.92 (0.83-0.96) | 0.81 (0.63-0.91) | 0.73 (0.49-0.87) | 0.53 (0.20-0.75) | 0.16 (0.00-0.50) | 0.97 (0.94-0.99) |
| nHF (ms^2^) | Resting | 0.98 (0.96-0.99) | 0.92 (0.84-0.96) | 0.89 (0.77-0.95) | 0.54 (0.20-0.76) | 0.36 (0.00-0.65) | 0.47 (0.12-0.72) | 0.98 (0.96-0.99) |
|  | Exercise | 0.99 (0.98-1.00) | 0.95 (0.89-0.98) | 0.83 (0.66-0.92) | 0.63 (0.33-0.81) | 0.44 (0.08-0.70) | 0.16 (0.00-0.50) | 0.99 (0.98-1.00) |
|  | Post-exercise recovery | 0.98 (0.96-0.99) | 0.90 (0.80-0.95) | 0.79 (0.60-0.90) | 0.71 (0.46-0.86) | 0.37 (0.00-0.65) | 0.13 (0.00-0.48) | 0.98 (0.96-0.99) |
| NN: normal-to-normal interval; AVNN: Average of NN; SDNN: standard deviation of NN; SDSD: standard deviation of successive difference of NN; RMSSD: root-mean-square of successive difference of NN; pNN50: percentage of adjacent NNs that differ from each other by more than 50 ms; TP: total power; VLF: very-low-frequency power of (0.0033–0.04) Hz; LF: low-frequency power of (0.04–0.15) Hz; HF: high-frequency power of (0.15–0.4) Hz; LF/HF: ratio of low-frequency power to high-frequency power; nLF: normalized low-frequency power (LF/(TP–VLF)); nHF: normalized high–frequency power (HF/(TP–VLF)). | | | | | | | | |
|  | | | | | | | | |

Table S3. The 50 % limits of agreement for ultra-short-term heart rate variability variables in each condition.

| HRV variable | Condition | Mean (95 % of confidence interval) | | | | | | |
| --- | --- | --- | --- | --- | --- | --- | --- | --- |
|  |  | 300 s | 240 s | 180 s | 120 s | 60 s | 30 s | 10 s |
| AVNN (ms) | Resting | 0 (0.00-0.01) | 0 (0.00-0.01) | 0.01 (0.00-0.02) | 0.01 (0.00-0.03) | 0.02 (0.00-0.04) | 0.03 (0.00-0.06) | 0 (0.00-0.01) |
|  | Exercise | 0.02 (0.01-0.03) | 0.04 (0.02-0.06) | 0.06 (0.04-0.08) | 0.07 (0.05-0.10) | 0.09 (0.06-0.11) | 0.09 (0.07-0.12) | 0.02 (0.01-0.03) |
|  | Post-exercise recovery | -0.01 (-0.01-0.00) | -0.02 (-0.03--0.01) | -0.04 (-0.06--0.01) | -0.08 (-0.12--0.05) | -0.13 (-0.17--0.09) | -0.18 (-0.23--0.13) | -0.01 (-0.01-0.00) |
| SDNN (ms) | Resting | 0 (-0.05-0.04) | -0.01 (-0.06-0.04) | -0.01 (-0.07-0.04) | -0.01 (-0.09-0.08) | -0.13 (-0.30-0.03) | -0.44 (-0.72--0.16) | 0 (-0.05-0.04) |
|  | Exercise | -0.12 (-0.20--0.05) | -0.32 (-0.48--0.15) | -0.48 (-0.68--0.28) | -0.62 (-0.90--0.34) | -0.71 (-0.97--0.45) | -0.92 (-1.18--0.65) | -0.12 (-0.20--0.05) |
|  | Post-exercise recovery | 0.02 (0.00-0.04) | 0.04 (-0.03-0.10) | 0.03 (-0.10-0.16) | -0.12 (-0.34-0.09) | -0.51 (-0.84--0.17) | -1.17 (-1.52--0.81) | 0.02 (0.00-0.04) |
| SDSD (ms) | Resting | 0.01 (-0.01-0.03) | 0.02 (0.00-0.05) | 0.04 (0.00-0.08) | 0.06 (-0.01-0.14) | 0.09 (-0.02-0.21) | 0.06 (-0.16-0.29) | 0.01 (-0.01-0.03) |
|  | Exercise | 0.04 (0.00-0.08) | 0.1 (0.03-0.17) | 0.15 (0.05-0.24) | 0.17 (0.05-0.29) | 0.2 (0.05-0.35) | 0.17 (-0.01-0.36) | 0.04 (0.00-0.08) |
|  | Post-exercise recovery | 0.01 (0.00-0.03) | 0.04 (0.00-0.08) | 0.06 (-0.02-0.15) | 0.01 (-0.15-0.17) | -0.18 (-0.41-0.06) | -0.49 (-0.78--0.21) | 0.01 (0.00-0.03) |
| RMSSD (ms) | Resting | 0.01 (-0.01-0.03) | 0.02 (0.00-0.05) | 0.04 (0.00-0.08) | 0.06 (-0.02-0.13) | 0.08 (-0.03-0.20) | 0.03 (-0.19-0.26) | 0.01 (-0.01-0.03) |
|  | Exercise | 0.04 (0.00-0.08) | 0.1 (0.03-0.17) | 0.15 (0.05-0.24) | 0.17 (0.04-0.29) | 0.19 (0.04-0.35) | 0.15 (-0.03-0.33) | 0.04 (0.00-0.08) |
|  | Post-exercise recovery | 0.01 (0.00-0.03) | 0.04 (-0.01-0.08) | 0.06 (-0.03-0.15) | 0.01 (-0.15-0.16) | -0.18 (-0.42-0.05) | -0.51 (-0.79--0.23) | 0.01 (0.00-0.03) |
| pNN50 (%) | Resting | 0.04 (-0.04-0.12) | -1 (-4.82-2.82) | -0.92 (-4.75-2.92) | -0.84 (-4.70-3.01) | -4.24 (-11.8-3.33) | -10.15 (-20.6-0.28) | 0.04 (-0.04-0.12) |
|  | Exercise | -1.03 (-4.97-2.92) | -0.87 (-4.85-3.10) | -5.1 (-13.26-3.05) | -8.32 (-17.9-1.21) | -9.38 (-19.4-0.59) | -13.98 (-24.7--3.27) | -1.03 (-4.97-2.92) |
|  | Post-exercise recovery | 0.08 (0.03-0.13) | 0.17 (0.03-0.31) | 0.27 (0.04-0.50) | -4.17 (-11.63-3.29) | -7.76 (-17.23-1.70) | -17.3 (-28.3--6.36) | 0.08 (0.03-0.13) |
| TP (ms^2^) | Resting | -0.03 (-0.13-0.07) | -0.03 (-0.21-0.15) | -0.02 (-0.31-0.27) | -0.11 (-0.47-0.25) | -0.65 (-1.20--0.10) | -1.83 (-2.70--0.96) | -0.03 (-0.13-0.07) |
|  | Exercise | -0.03 (-0.15-0.09) | -0.02 (-0.26-0.22) | -0.21 (-0.54-0.13) | -0.44 (-0.96-0.07) | -0.75 (-1.39--0.12) | -1.81 (-2.38--1.24) | -0.03 (-0.15-0.09) |
|  | Post-exercise recovery | 0.01 (-0.12-0.14) | 0.02 (-0.24-0.28) | -0.17 (-0.59-0.24) | -0.62 (-1.25-0.01) | -1.09 (-1.80--0.37) | -2.56 (-3.44--1.69) | 0.01 (-0.12-0.14) |
| VLF (ms^2^) | Resting | -0.05 (-0.19-0.09) | -0.13 (-0.47-0.20) | -0.25 (-0.76-0.26) | -0.67 (-1.41-0.07) | -2.36 (-3.82--0.90) | -4.2 (-5.41--3.00) | -0.05 (-0.19-0.09) |
|  | Exercise | -0.12 (-0.29-0.05) | -0.22 (-0.61-0.18) | -0.57 (-1.14-0.00) | -1.42 (-2.24--0.61) | -2.52 (-3.46--1.58) | -4.48 (-5.55--3.41) | -0.12 (-0.29-0.05) |
|  | Post-exercise recovery | 0.06 (-0.11-0.22) | 0.09 (-0.25-0.44) | -0.27 (-0.82-0.28) | -1.33 (-2.05--0.62) | -2.83 (-3.70--1.96) | -5.4 (-6.61--4.18) | 0.06 (-0.11-0.22) |
| LF (ms^2^) | Resting | -0.01 (-0.14-0.12) | 0.03 (-0.23-0.30) | 0.04 (-0.36-0.44) | 0.15 (-0.38-0.67) | -0.39 (-1.08-0.30) | -2.37 (-3.44--1.29) | -0.01 (-0.14-0.12) |
|  | Exercise | 0.11 (-0.01-0.23) | 0.18 (-0.08-0.45) | 0.15 (-0.23-0.52) | 0.11 (-0.53-0.74) | -0.09 (-0.79-0.60) | -1.93 (-2.74--1.11) | 0.11 (-0.01-0.23) |
|  | Post-exercise recovery | -0.05 (-0.18-0.08) | -0.12 (-0.43-0.18) | -0.13 (-0.65-0.40) | -0.33 (-1.06-0.40) | -0.61 (-1.49-0.27) | -2.87 (-3.90--1.84) | -0.05 (-0.18-0.08) |
| HF (ms^2^) | Resting | 0.03 (-0.03-0.08) | 0.03 (-0.16-0.21) | 0.05 (-0.21-0.30) | 0.1 (-0.28-0.48) | 0.13 (-0.41-0.67) | -0.33 (-1.27-0.61) | 0.03 (-0.03-0.08) |
|  | Exercise | 0.11 (0.01-0.21) | 0.17 (-0.03-0.36) | 0.16 (-0.14-0.46) | 0.37 (-0.07-0.82) | 0.3 (-0.18-0.79) | 0.06 (-0.51-0.63) | 0.11 (0.01-0.21) |
|  | Post-exercise recovery | 0.09 (0.00-0.19) | 0.26 (0.07-0.45) | 0.4 (0.02-0.78) | 0.3 (-0.33-0.94) | 0.06 (-0.79-0.90) | -0.75 (-1.66-0.16) | 0.09 (0.00-0.19) |
| LF/HF (n.u.) | Resting | 0.03 (-0.06-0.11) | 0.05 (-0.08-0.18) | 0.04 (-0.15-0.24) | 0.11 (-0.12-0.35) | -0.11 (-0.41-0.19) | -1.01 (-1.46--0.55) | 0.03 (-0.06-0.11) |
|  | Exercise | 0.08 (-0.04-0.20) | 0.11 (-0.06-0.28) | 0.15 (-0.08-0.39) | 0.13 (-0.15-0.41) | 0.05 (-0.25-0.35) | -0.81 (-1.41--0.21) | 0.08 (-0.04-0.20) |
|  | Post-exercise recovery | -0.02 (-0.17-0.13) | -0.06 (-0.36-0.24) | 0.03 (-0.31-0.37) | 0.07 (-0.26-0.40) | 0.03 (-0.47-0.52) | -0.84 (-1.41--0.28) | -0.02 (-0.17-0.13) |
| nLF (ms^2^) | Resting | 0.07 (-0.04-0.18) | 0.05 (-0.16-0.25) | 0.05 (-0.14-0.23) | 0.06 (-0.39-0.52) | 0.41 (-0.10-0.91) | 1.03 (0.62-1.45) | 0.07 (-0.04-0.18) |
|  | Exercise | 0.08 (-0.01-0.18) | 0.09 (-0.10-0.28) | 0.17 (-0.12-0.45) | 0.4 (0.02-0.78) | 0.45 (-0.13-1.03) | 1.17 (0.67-1.68) | 0.08 (-0.01-0.18) |
|  | Post-exercise recovery | 0.13 (0.00-0.25) | 0.33 (0.13-0.53) | 0.56 (0.27-0.85) | 0.7 (0.35-1.05) | 0.69 (0.23-1.16) | 1.27 (0.78-1.77) | 0.13 (0.00-0.25) |
| nHF (ms^2^) | Resting | -0.04 (-0.17-0.09) | 0.01 (-0.27-0.29) | 0 (-0.33-0.33) | 0.05 (-0.59-0.68) | -0.52 (-1.27-0.24) | -2.04 (-2.72--1.36) | -0.04 (-0.17-0.09) |
|  | Exercise | 0 (-0.09-0.09) | 0.02 (-0.22-0.25) | -0.01 (-0.40-0.37) | -0.27 (-0.77-0.24) | -0.4 (-1.12-0.33) | -1.98 (-2.85--1.12) | 0 (-0.09-0.09) |
|  | Post-exercise recovery | -0.14 (-0.26--0.03) | -0.39 (-0.67--0.11) | -0.53 (-0.96--0.10) | -0.63 (-1.12--0.14) | -0.67 (-1.48-0.15) | -2.12 (-2.96--1.27) | -0.14 (-0.26--0.03) |
| NN: normal-to-normal interval; AVNN: Average of NN; SDNN: standard deviation of NN; SDSD: standard deviation of successive difference of NN; RMSSD: root-mean-square of successive difference of NN; pNN50: percentage of adjacent NNs that differ from each other by more than 50 ms; TP: total power; VLF: very-low-frequency power of (0.0033–0.04) Hz; LF: low-frequency power of (0.04–0.15) Hz; HF: high-frequency power of (0.15–0.4) Hz; LF/HF: ratio of low-frequency power to high-frequency power; nLF: normalized low-frequency power (LF/(TP–VLF)); nHF: normalized high-frequency power (HF/(TP–VLF)). | | | | | | | | |
|  | | | | | | | | |
